# Addictive Potential of e-Cigarettes as Reported in e-Cigarette Online Forums: Netnographic Analysis of Subjective Experiences

**DOI:** 10.2196/41669

**Published:** 2023-01-06

**Authors:** Daria Szafran, Tatiana Görig, Sabine Vollstädt-Klein, Nadja Grundinger, Ute Mons, Valerie Lohner, Sven Schneider, Marike Andreas

**Affiliations:** 1 Division of Public Health, Social and Preventive Medicine Center for Preventive Medicine and Digital Health Medical Faculty Mannheim, Heidelberg University Mannheim Germany; 2 Department of Medical Informatics, Biometry and Epidemiology Friedrich-Alexander-Universität Erlangen-Nürnberg Erlangen Germany; 3 Department of Addictive Behavior and Addiction Medicine Central Institute of Mental Health, Medical Faculty of Mannheim University of Heidelberg Mannheim Germany; 4 Mannheim Center for Translational Neurosciences Medical Faculty of Mannheim University of Heidelberg Mannheim Germany; 5 Cardiovascular Epidemiology of Aging Department of Cardiology Faculty of Medicine and University Hospital Cologne, University of Cologne Cologne Germany

**Keywords:** e-cigarette, addiction, netnographic analysis, smoking, tobacco, substance use, vaping

## Abstract

**Background:**

While e-cigarettes usually contain nicotine, their addictive potential is not yet fully understood. We hypothesized that if e-cigarettes are addictive, users will experience typical symptoms of addiction.

**Objective:**

The aim of our study was to investigate whether and how e-cigarette users report signs of addiction.

**Methods:**

We identified 3 large German-language e-cigarette online forums via a systematic Google search. Based on a netnographic approach, we used deductive content analysis to investigate relevant posts in these forums. Netnography has the advantage of limiting the social desirability bias that prevails in face-to-face research, such as focus groups. The data were coded according to the Diagnostic and Statistical Manual of Mental Disorders, 5th Edition (DSM-5) criteria for tobacco use disorder, adapted for e-cigarettes. The DSM-5 criteria were used to portray a broad spectrum of possible experiences of addiction.

**Results:**

Overall, 5337 threads in 3 forums were screened, and 451 threads containing relevant information were included in the analysis. Users reported experiences consistent with the DSM-5 criteria, such as craving e-cigarettes, excessive time spent vaping, and health issues related to e-cigarette use. However, our analysis also showed that users reported the absence of typical tobacco use disorder criteria, such as successful attempts to reduce the nicotine dosage. For most themes, reports of their absence were more frequent than of their presence. The absence of perceived addiction was mostly reported in contrast to prior tobacco smoking.

**Conclusions:**

This is the first study to use a netnographic approach to explore unfiltered self-reports of experiences of e-cigarette addiction by users in online forums. As hypothesized, some but not all users reported subjective experiences that corresponded to the criteria of tobacco use disorder as defined by the DSM-5. Nevertheless, subjective reports also indicated that many e-cigarette users felt in control of their behavior, especially in contrast to their prior use of tobacco cigarettes. The finding that some e-cigarette users subjectively experience addiction highlights the need for effective cessation programs to support users who experience their e-cigarette use as burdensome. This research can guide the refinement of instruments to assess e-cigarette addiction and guide cessation programs.

**International Registered Report Identifier (IRRID):**

RR2-10.1186/s40359-021-00682-8

## Introduction

Electronic cigarettes, or e-cigarettes, are handheld electronic devices that heat a liquid to create vapor that users inhale [1]. Although they were introduced to global markets relatively recently, e-cigarettes have become increasingly popular, with an estimated 68 million users globally in 2020 [2]. Many e-cigarette users treat vaping as a hobby and share their experiences on online forums [3,4], social media platforms [5-7], and conventions [8].

Although e-cigarettes are often marketed as a less harmful alternative to tobacco [9-11], scholars highlight the inconsistency of findings regarding health- and addiction-related outcomes of e-cigarette use [12]. E-cigarettes usually contain nicotine, which is known to cause dependency [13], and the World Health Organization classifies them as “addictive and not without harm” [1]. However, more research is needed to explore e-cigarettes’ addictive potential.

For this paper, substance-related addiction was defined as a “recurring desire to continue taking the drug despite harmful consequences” [14]. Current models of addiction [15] highlight 3 aspects central to the development and maintenance of addiction: craving, tolerance, and withdrawal. These are also reflected in the 11 criteria for tobacco use disorder in the Diagnostic and Statistical Manual of Mental Disorders, 5th Edition (DSM-5) [16]. Craving is defined as the conscious experience of a desire to consume a substance [17]. Tolerance is demonstrated by either “a need for markedly increased amounts of tobacco to achieve the desired effect” or “a markedly diminished effect with continued use of the same amount of tobacco” [16]. Withdrawal is present when at least 4 symptoms (ie, irritability, frustration, anger, or unrest) occur and are perceived as distressing after abrupt cessation following daily use for at least several weeks [16]. Whether these symptoms are also applicable to e-cigarette use has not yet been fully clarified.

Previous studies provide some evidence for e-cigarettes’ addictive potential. Browne and Todd [18] found that users tended to increase their nicotine dosage and consume higher levels of nicotine over time, which indicates the development of tolerance. Liu et al [19] observed that—although tobacco cigarette smokers report stronger addiction symptoms—e-cigarette users’ “dependence on their product is not negligible,” and the majority perceived themselves as “addicted.” In a study conducted by Hughes et al [20], e-cigarette users who abstained from use for a week reported typical cravings, as well as several withdrawal symptoms. Apart from the mentioned symptoms, the DSM-5 classifies the use of a substance despite health problems as a criterion for substance use disorder. While there is some evidence linking e-cigarette use to negative health effects, such as coughing and nausea [21], no study exists that shows how such symptoms affect e-cigarette consumption behavior. To the best of our knowledge, there is no evidence of e-cigarette users meeting other criteria for tobacco use disorder defined by the DSM-5.

Given this research gap, we hypothesized that if e-cigarette consumption led to addiction, users would report signs of tobacco use disorder. Thus, the aim of our study was to shed light on whether and how e-cigarette users subjectively perceive typical signs of addiction according to the DSM-5 criteria for tobacco use disorder. To answer this question within the Evaluation of the Addictive Potential of e-Cigarettes (EVAPE) project, we used a netnographic approach [22] and analyzed current data from German-language online forums on e-cigarettes.

## Methods

### Study Design and Setting

The study design was based on a netnographic approach, which applies ethnographic techniques to online social interactions. This approach was first developed and applied in a sociological research context and is increasingly used in epidemiology and health research [23].

Previous research has shown that online forums are important spaces for e-cigarette users to communicate about relevant topics with like-minded people [24,25]. Within the forums, users communicate via threads, that is, online conversations consisting of connected messages on a specific topic sent by different users over a flexible time period. Discussion threads develop independently of the researcher. Users can anonymously talk about sensitive issues in such forums with less fear of social repercussions [26]. The main reason for choosing to examine these forums was to obtain more authentic perceptions about e-cigarette use than we could have obtained from face-to-face-methods. In this context, netnography can provide insights into health and dependence-related experiences without social desirability bias [27]. Based on these considerations, we decided to analyze German-language online forums on e-cigarettes in this study. Both authors involved in the analysis (TG and DS) are experienced and trained in qualitative methodologies. The methodology and results are reported according to the Consolidated Criteria for Reporting Qualitative Research (COREQ) [28]. The COREQ checklist is provided in Multimedia Appendix 1.

### Data Collection

We used a 3-step procedure to collect the data for this study. First, 1 study team member (DS) conducted a search for the relevant online forums via Google search using different combinations of the German versions of the keywords “e-cigarette” AND “(online) forum” (Multimedia Appendix 2). A search on February 18, 2021, revealed 76 online forums containing threads on e-cigarettes. To select forums for the final analysis, we used the following criteria: (1) e-cigarettes were the forum’s main topic, (2) the forum users communicated in German, (3) the forum was publicly accessible (ie, no registration was required to read the user contributions), (4) the forum was active in the 4 weeks prior to February 18, 2021, (5) there were at least 500,000 posts in the forum (to ensure user anonymity), (6) there were at least 5000 forum members, (7) there was a search function for the forum, (8) there was no affiliation with the tobacco industry, and (9) there was a public access disclaimer in the terms and conditions. Overall, 3 forums met these criteria, 2 from Germany and 1 from Switzerland.

In the second step, we searched the identified forums for previously defined keywords. For each criterion, we defined 1 to 3 keywords that described the respective theme as accurately as possible (Multimedia Appendix 3). The initial keyword search in all 3 forums returned 5337 threads (the last search was on April 9, 2021). The threads widely differed in length, with some only having a few posts and others containing hundreds of posts authored by dozens of users over the span of months.

In the third step, 2 team members (DS and TG) screened all the threads, which were posted between April 9 and June 18, 2021. To assess whether a thread was relevant to the theme, we viewed short threads in their entirety. For long threads (≥5 pages), we based the content assessment on the title of the thread and the post in which the keyword was mentioned. Threads without posts on relevant themes were excluded at this stage. We excluded posts from users who explicitly reported also smoking conventional cigarettes (dual users) from our analysis.

### Data Analysis

We chose a deductive thematic approach, as described by Mayring [29], as the method of analysis. The themes were based on the DSM-5 criteria for tobacco use disorder, which we adapted for e-cigarettes (Table 1). We chose this method as the DSM-5 criteria give a comprehensive overview of different symptoms of addiction that are internationally accepted. Deductive analysis allowed us to explore whether such symptoms were also perceived by e-cigarette users.

After data piloting, we decided to use positive (ie, a sign was reported) and negative (ie, the opposite was reported) coding for each theme. For example, a forum user could report a craving for e-cigarettes (a positive code), or an absence of craving (a negative code). The program Maxqda (version 20.3.0; Verbi GmbH) was used to code the data. According to the research standards for our deductive thematic approach [29], we designed a coding manual before conducting the qualitative content analysis. Two team members (TG and DS) piloted the data analysis with 25 threads and double-coded 235 threads. The intercoder agreement was 69.7% for the 235 threads. Disagreements were discussed and resolved by consensus between the 2 authors in each case. When agreement could not be reached, a third team member was consulted, who made the final decision (SS). Based on the discussions, we adapted and finalized the coding manual. After this, TG and DS analyzed the remaining threads independently.

**Table 1:** Diagnostic and Statistical Manual of Mental Disorders, 5th Edition criteria for tobacco use disorder, modified for e-cigarettes.

Criterion	Wording for tobacco addiction	Wording for e-cigarette addiction
1	Tobacco is often taken in larger amounts or over a longer period than was intended.	E-cigarettes are often used with greater nicotine dosage or for longer than intended.
2	There is a persistent desire or unsuccessful efforts to cut down or control tobacco use.	There is a persistent desire or unsuccessful efforts to reduce or control e-cigarette use.
3	A great deal of time is spent in activities necessary to obtain or use tobacco.	A great deal of time is spent in activities necessary to obtain or use e-cigarettes or e-cigarette equipment.
4	Craving, or a strong desire or urge to use tobacco.	Craving, or a strong desire or urge to use e-cigarettes.
5	Recurrent tobacco use resulting in a failure to fulfill major role obligations at work, school, or home (e.g., interference with work).	Repeated e-cigarette use resulting in failure to fulfill important responsibilities at work, school, or home (e.g., interference with work).
6	Continued tobacco use despite having persistent or recurrent social or interpersonal problems caused or exacerbated by the effects of tobacco (e.g., arguments with others about tobacco use).	Continued e-cigarette use despite ongoing or repeated social or interpersonal problems caused or exacerbated by the effects of e-cigarettes (e.g., arguing with others about e-cigarette use).
7	Important social, occupational, or recreational activities are given up or reduced because of tobacco use.	Important social, professional, or recreational activities are abandoned or curtailed because of e-cigarette use.
8	Recurrent tobacco use in situations in which it is physically hazardous (e.g., smoking in bed).	Recurrent e-cigarette use in which it is physically hazardous (e.g., e-cigarette use in bed).
9	Tobacco use is continued despite knowledge of having a persistent or recurrent physical or psychological problem that is likely to have been caused or exacerbated by tobacco.	Continued e-cigarette use despite knowledge of a persistent or recurring physical or psychological problem that is likely caused or exacerbated by e-cigarettes.
10	Tolerance, as defined by either of the following: a. A need for markedly increased amounts of tobacco to achieve the desired effect. b. A markedly diminished effect with continued use of the same amount of tobacco.	Tolerance, as defined by any of the following: a. A desire for a marked increase in e-cigarette dosage to achieve the desired effect. b. A significantly reduced effect with continued use of the same e-cigarette dosage.
11	Withdrawal, as manifested by either of the following: a. The characteristic withdrawal syndrome for tobacco (refer to Criteria A and B of the criteria set for tobacco withdrawal). b. Tobacco (or a closely related substance, such as nicotine) is taken to relieve or avoid withdrawal symptoms.	Withdrawal symptoms, as manifested by any of the following: a. Characteristic withdrawal syndrome related to e-cigarettes (irritability, frustration, anger, anxiety, difficulty concentrating, increased appetite, restlessness, depressed mood, insomnia). b. E-cigarettes (or nicotine in the case of nicotine-containing e-cigarettes) are used to relieve or avoid withdrawal symptoms.

Quotes chosen as examples for this paper were paraphrased and translated to English by 1 author (MA) and checked for accuracy by 2 authors (DS and SS).

In addition to the qualitative analysis, we collected quantitative data on the frequency of reported signs.

### Ethical Considerations

Specific ethical aspects need to be considered when researching online communities [30]. Since we did not participate in forum discussions, it was not necessary to inform users that their posts were used for research. Users were made aware of the public accessibility of their posts by the forums’ terms and conditions. Additionally, we deliberately selected forums that had a high number of active users to ensure their anonymity in the reporting of the results. Finally, we used a random number generator to identify users, and no further individual user information was documented. This procedure is in line with discussions on ethics in online research [30] and other research in a similar context [24]. The Medical Ethics Committee of the Medical Faculty Mannheim of the University of Heidelberg approved this procedure (2017-567-N-MA). The study design was preregistered on the Open Science Framework [31] and has been described in a previous publication [32].

## Results

### Overall Results

Frequently discussed topics in the forums were e-cigarette types and setups, different kinds of liquids and flavors, and general lifestyle. The forums were active, with the biggest forum having 1,458,441 posts and approximately 400 daily new posts by 14,751 users (forum statistics at time of data extraction in February 2021). The users’ nicknames, as well as their posts, indicated that the majority were male and had smoked tobacco cigarettes before their e-cigarette use. As described above, we excluded posts from users who explicitly reported being current dual users from our analysis.

**Figure 1:**
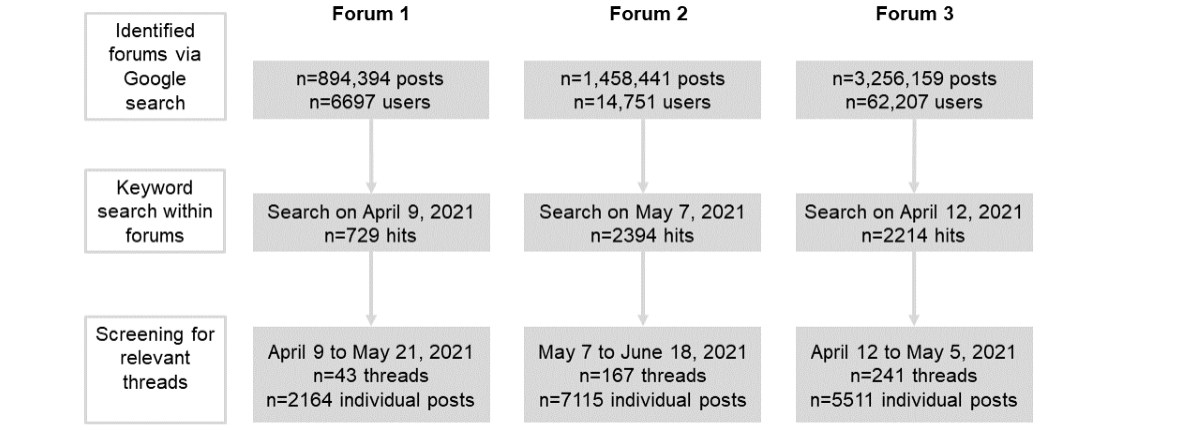
Flowchart of searches on the forums. Values for total posts and number of users in the top row reflect searches made on February 18, 2021.

Of the 5337 threads initially screened in the 3 forums, 451 contained relevant information regarding addiction and were included in the data analysis (Figure 1). We identified posts on 9 of 11 themes based on the DSM-5 criteria. In the following sections, we present each theme individually, sorted by the frequency of total mentions. Table 2 shows the frequency of user reports. Table 3 shows representative quotes for each theme.

**Table 2:** Frequency of user reports. Themes are based on the Diagnostic and Statistical Manual of Mental Disorders, 5th Edition criteria for substance use disorder.

Themes	Reports of presence, n	Reports of absence, n
Unsuccessful attempts to reduce	731	31
Expenditure of time	296	0
Health problems	91	169
Craving	29	93
Withdrawal	18	31
Social problems related to use	11	22
Tolerance	7	1
Loss of control	3	1
Restriction of social activities	1	0
Neglecting responsibilities	0	0
Hazardous use	0	0

**Table 3:** Summary of the criteria for e-cigarette addiction and examples of their presence and absence.

Criterion	Themes	Example reports of presence	Example reports of absence
1	Loss of control	“I’m actually annoyed by my vaping. I vape quite a lot and reach for my e-cigarette almost automatically.”	“I vape 18mg just to avoid vaping constantly.”
2	Unsuccessful attempts to reduce	“I increased the nicotine from 0.5 to 2mg because it was not possible to go with less or no nicotine for me.”	“I started off with a 6mg dose but decreased it daily. At one point I vaped less than 6mg and then I could live with no nicotine at all. I also think without nicotine the liquid tastes best.”
3	Expenditure of time	“When I’m at home I vape constantly, and when I leave the house I always bring more than one vape with me.”	—^a^
4	Craving	“When I imagine not being able to use my e-cigarette, I get anxious and instantly have to vape.”	“I once went to a vaper meeting and went the whole evening without my e-cigarette. No one noticed and I did not feel craving and had such a good evening.”
6	Social problems related to use	“My wife doesn’t like it when I vape. When I vape in the house she complains or looks disgusted.”	“My manager and I discussed vaping at work and he is okay with it as long as no one complains. Some of my colleagues think my office smells really good because of the e-cigarette.”
7	Restriction of social activities	“Since I spend some money on my hardware, I had to stop my hobby for the time being.”	—
9	Health problems	“What I find very annoying is that I constantly feel like I have a cough. It feels like an allergy, which means I sneeze often, and I cough green mucus in the mornings.”	“Since I transitioned to the e-cigarette my mental and physical health has improved significantly. I do more sports and my lungs and heart don’t complain anymore when I work out. I used to get panic attacks and was super nervous but not anymore!”
10	Tolerance	“As you know, I have been using an e-cigarette for a bit more than a month now. Now, I do not get a buzz from vaping anymore. So, I have put more nicotine into my mix again.”	“I am noticing that an increased nicotine dosage has negative effects...I have trouble sleeping, a higher blood pressure, palpitations and so on.”
11	Withdrawal	“I often work several hours without a break and cannot vape during that time. Sometimes I get quite angry and my lung complains.”	“I believe I could stop vaping anytime. I actually was able to stop smoking more than once. It did not affect me at all, I did not get nervous or anything. In contrast to when I smoked cigarettes, I am now able to concentrate on long books without vaping.”

^a^Not available. Reports of absence for these themes were not found and examples were not coded.

### Unsuccessful Attempts to Reduce

Attempts to reduce the nicotine dosage were frequently discussed in the forums. User #12 described their unsuccessful attempt: “I started off with a lower nicotine dosage but I did not get a buzz from it. So now I vape a higher dosage and don’t want to go back.” On the other hand, more users related successful attempts to reduce the amount they consume. User #398 reported, “I was a heavy smoker but when I started vaping I was able to reduce from 18mg to 7mg and have been vaping this mix for some years now.”

### Expenditure of Time

Many users reported almost uninterrupted e-cigarette use throughout the day. This behavior was often subjectively perceived as being deliberately chosen. For instance, user #137 wrote, “I vape incessantly, in the morning, when I work, on the way to work, and when I go out in the evening.” Other users stated that they spent a great deal of time consuming in specific situations, such as while drinking alcohol.

We did not code a negative code for this theme, because the only relevant post mentioning a low time expenditure fitted the theme “loss of control” more accurately.

### Health Problems

Different posts showed that even though users experienced health problems, they continued to use e-cigarettes. User #10 wrote, “I get a swelling under my eyes whenever I vape more than usual. A few days after that I can still feel the swelling in my cheeks as well as under the eyes.” Other symptoms described by users were headaches, inflammation, rash, swelling, dizziness, and digestive problems. Moreover, problems were reported to be associated with specific liquids and nicotine dosages. The ingredients propylene glycol and vegetable glycerin were prominently discussed as reasons for allergic reactions and other health issues.

In contrast, other users perceived that their physical health improved after they switched from tobacco to e-cigarettes. User #71 reported, “My quality of life really improved when I stopped normal [ie, tobacco] smoking. When working out I breathe more easily and I can taste and smell much better.” Moreover, improvements in mental health were also described. User #85 said that their panic attacks had stopped (“I used to get panic attacks and was super nervous but not anymore!”) since switching to an e-cigarette, and user #36 related that they slept better: “I wake up before my alarm clock.” No user reported improvements in health due to e-cigarette use without describing a contrast with tobacco.

### Craving

Some users reported explicit cravings for e-cigarettes, such as frequent thoughts about e-cigarettes or seeking out favorable opportunities to use e-cigarettes. Descriptions of craving in the context of specific situations or habits were also found. For example, user #54 reported, “There are specific moments where I feel craving for my e-cigarette. Mostly when I’m alone or when I had a really exhausting day I want to reward myself with vaping.” Another user stated, “Honestly, I am addicted to vaping. Wherever I go, be it my office or in my free time, I instantly look for a place where I can vape.”

On the other hand, more users described a weak or missing desire for e-cigarette use. For these users, it seemed easy to abstain, regardless of the situation. User #282 explained, “When I still smoked I would always go for a smoke after lunch. But now I can sit and relax after lunch and enjoy the time.”

### Withdrawal

Users described withdrawal symptoms when they could not use e-cigarettes for a long time or when the nicotine dosage was subjectively too low. Withdrawal symptoms such as physical unrest, nervousness, irritability, and difficulty concentrating were specified. For instance, user #57 reported, “I was using a 18mg dosage. I was very confused why I was becoming more aggressive and hyperactive. And then I realized it was because I was vaping nicotine-free for half the day.”

On the other hand, users also reported the absence of withdrawal symptoms, even when it was not possible to use e-cigarettes for a long time or when they used a lower nicotine dosage. For example, user #7 said, “While I work, I cannot vape for the whole day. But that’s fine by me.”

### Social Problems Related to Use

Users reported (1) breaking off contact with people in whose presence e-cigarette use was undesired, (2) experiencing negative reactions from people in their immediate environment (family, friends, and colleagues) to e-cigarettes, and (3) experiencing situations that escalated due to personal e-cigarette use. For example, user #493 wrote, “Once I started vaping, my circle of friends changed. I broke off contact with those who do not tolerate vaping when we meet.”

Especially in contrast to when they used tobacco cigarettes, some users reported that reactions within their social environment to their e-cigarette use were positive. For example, user #41 related, “In the beginning my friends and family were worried. But now they see how much better my health is, they don’t see it as a problem.” Users also frequently reported being complimented on certain liquid flavors by their friends or colleagues.

### Tolerance

When discussing their dosage and liquid mixtures, some users recounted the development of tolerance. This means that their “buzz” or nicotine high ceased, causing them to increase their dosage. For example, user #89 admitted, “I have really become increasingly tolerant.” Another user (user #11) asked the forum, “I don’t miss cigarettes but lately I have been vaping much more than usual and I do not get a buzz anymore. Am I the only one who gets more craving after vaping for some months?”

Only one user (user #51) described negative physical reactions to the usual nicotine dose: “I am realizing the effects of more nicotine...I have trouble sleeping, higher blood pressure, palpitations and so on.”

### Loss of Control

A few users revealed that they used their e-cigarettes almost continuously, even though they did not intend to. As a consequence, they subjectively perceived this behavior as a problem or burden. For example, user #355 described themselves as a *Dauernuckler*, the German word for someone who is constantly sucking on their e-cigarette, and admitted, “I’m actually very annoyed by it but I can’t stop.”

In contrast, only one user reported that they managed to control the amount they vaped by keeping a constant nicotine level: “I vape 18mg just to avoid vaping constantly.”

### Restriction of Social Activities

One user (user #243) reported they had to give up an activity because of e-cigarette use: “Since I spend some money on my setup, I had to stop my hobby for the time being.” We did not code a negative code for criterion 7 in cases when users saw e-cigarette use as their new hobby, since we did not interpret substance consumption as a new social activity.

## Discussion

### Principal Findings

This study is the first to show that e-cigarette users report subjective experiences of addiction based on the DSM-5 criteria for tobacco use disorder. This was the result of a netnographic analysis of 451 threads from 3 large German-language online forums. In summary, some but not all users reported subjective experiences that corresponded to the criteria of a tobacco use disorder as defined by the DSM-5. Our aim was not to diagnose forum members but to use the DSM criteria to explore subjective experiences of addiction. Our chosen method of accessing the field allowed us to take an unfiltered look at user reports. Our findings are consistent with previous qualitative and quantitative research on e-cigarette addiction, as discussed below.

The criterion we coded positively most often was “expenditure of time.” While using any substance involves a certain amount of time, an excessive preoccupation with the consumption process is an important aspect of the definition of addiction. Indeed, specific questions regarding time are included in instruments such as the Fagerström test of e-cigarette dependence [33].

Furthermore, our analysis showed that many users continued their e-cigarette use despite experiencing health problems such as chest pain, skin swelling, or gastrointestinal symptoms. This reflects clinical observations of e-cigarette users’ health [34]. Similarly, a content analysis of the social media site Reddit demonstrated that subjective health problems were the most common reason for e-cigarette users to quit [4]. Thus, it seems that health problems are a central aspect of negative experiences related to vaping and might therefore be an important motivator for vaping cessation.

Moreover, we found that users reported experiencing cravings and withdrawal when they were not able to use e-cigarettes. This result confirms longitudinal studies conducted in the United States [19,35]. Like our study, a clinical trial demonstrated that e-cigarette users experienced withdrawal symptoms similar to the DSM-5 criteria when they were asked to stop their use for a week [20]. Cravings and withdrawal symptoms thus seem to be significant signs of addiction in e-cigarette users.

In the forums we investigated, some users described unsuccessful attempts to reduce the amount of nicotine they consumed. Indeed, they reported increasing their nicotine dosage over time. As of yet, only one study exists on nicotine dosing patterns in e-cigarette users over time, by Browne and Todd [18], who showed that e-cigarette users who had vaped for a greater number of years used a higher nicotine dosage. We also found that some users reported developing tolerance to the nicotine dosage of their e-cigarettes and seeking a nicotine “buzz.” Few studies have investigated tolerance in e-cigarette users. In a qualitative study, teenagers reported taking “tolerance breaks” to feel a nicotine buzz [36]. However, in contrast to the other themes, we found this theme less frequently.

Nevertheless, we also found that the absence of signs of addiction according to the DSM-5 criteria was frequently reported by users. This especially applied to the theme “attempts to reduce.” The majority of posts coded for this theme described successful attempts to reduce either e-cigarette use or nicotine dosage. One reason for this could be that in the forums, experienced members recommended starting with a high nicotine dosage when switching from tobacco to e-cigarettes, in order to avoid relapse. Furthermore, more posts discussed an absence of craving for e-cigarettes than its presence. The same was true for the themes “health problems,” “social problems related to use” and “withdrawal” but with less pronounced differences in frequencies. Importantly, we found that an absence of these experiences was mostly reported in contrast to the previous use of tobacco cigarettes. Likewise, Schilling et al [24] reported that the health threats of e-cigarette smoking during pregnancy were perceived as relative risks in online forums, indicating that they were seen as dangerous, but not as much so as tobacco cigarettes. This framing of e-cigarettes as a counterpart to tobacco cigarettes is best summed up by Bell and Keane [37]: “It is in comparison with smoking that vaping comes to represent health, happiness and freedom.”

Regarding social acceptability, our analysis showed that while some users did experience social problems related to use, e-cigarette use was mostly accepted in their environments, especially in contrast to smoking. Users also reported using their e-cigarettes in different locations, such as their offices. This reflects previous research indicating that e-cigarette use is seen as more socially acceptable than smoking and is therefore possible in spaces in which smoking is restricted or undesirable [38].

Finally, we did not find any posts corresponding to the DSM-5 criteria “neglecting responsibilities” or “hazardous use.” This finding is in line with an ongoing debate on the appropriateness of the DSM-5 criteria for tobacco use disorder. Sokya and Baumgärtner [39] criticized some of the criteria for being applicable to illicit drugs but not to tobacco, as cigarettes are more easily available, more acceptable, and less hazardous than most “hard drugs.” In addition, this study and recent research suggests that the criteria for tobacco and e-cigarette addiction might differ substantially [40]. Our netnographic approach indicates that criteria such as tolerance or loss of control might be less relevant or less applicable to e-cigarette users.

In sum, our study offers a comprehensive and broad overview of subjective experiences of addiction, adding to the existing literature, which so far has mostly focused on specific symptoms. Our results indicate that some users perceive e-cigarettes to be highly addictive and reflect on their incessant use and craving. Since our research indicates which experiences are perceived as burdensome by users, this knowledge could be a starting point for the refinement of instruments to measure e-cigarette addiction, as well as tools to identify users who are at risk for e-cigarette addiction. Even though e-cigarettes are perceived as less addictive or harmful than tobacco cigarettes by users, our study clearly demonstrates that users do experience addiction. Hence, more research on effective strategies for e-cigarette cessation and efforts to support users with effective cessation programs are required.

The evidence that there are users who experience signs of addiction is a piece of the overall puzzle of e-cigarette dependence. The interdisciplinary EVAPE project aims to provide further valuable insights based on neurobiological and longitudinal epidemiological data, as well as focus groups with e-cigarette users [32].

### Limitations

This study has some limitations. First, we relied on user self-reports. A systematic review demonstrated that self-reports of smoking are less accurate than biological measures, as smokers underreport their behavior [41]. Furthermore, our data were based on a highly specific group of e-cigarette users. While our study did not aim to find prevalent or representative users, it is important to consider that the overall positive stance of the forum users toward e-cigarettes could have had a further effect on the underreporting of signs of dependence.

Another limitation is the possibility that the analysis included posts by users who use e-cigarettes in addition to tobacco (ie, dual users). While we excluded posts containing explicit mentions of dual use, we could not exclude all posts by dual users. Since dual users have reported higher levels of addiction than exclusive e-cigarette users in previous research [42], this should be considered in the interpretation of the results. Moreover, we could not differentiate between users who used liquids with or without nicotine, and in the case of the former, we do not have data on dosage. Finally, a few threads relevant to our research question, especially on health topics, were not openly accessible, which could potentially have biased our results.

This study is the first to use a netnographic approach to explore self-reported signs of dependence in e-cigarette users. In contrast to other forms of qualitative research, such as interviews or focus group discussions, netnography approaches in the context of research on online forums have had to rely on limited responses, with no opportunity to clarify meanings, and hence bear the risk of misinterpreting responses. On the other hand, this approach avoids biases related to personal or group interviews, such as interviewer bias or social desirability bias [43]. However, there is still a risk of bias, as research has shown that forum users carefully choose the information they are willing to share to create a favorable image of themselves [44]. Our method did not allow us to identify statements that were adjusted by users as a result of their wish to please the online community. Future work should use focus groups and validated questionnaires to further explore experiences of addiction in e-cigarette users.

### Conclusion

The netnographic approach used in this study allowed us to explore unfiltered self-reports of addiction by e-cigarette users in online forums. Some but not all users reported subjective experiences that corresponded with the DSM-5 tobacco use disorder criteria. Nevertheless, we also found that users discussed the absence of such signs, and in most cases, reports of absence were more frequent than reports of presence. While it is not possible to derive a clinically reliable diagnosis of tobacco use disorder from such self-reports, our study clearly showed that some users reported signs that were comparable to the criteria for tobacco use disorder in the DSM-5.

## Appendix

Multimedia Appendix 1 COREQ checklist.

Multimedia Appendix 2 Search terms of online search for e-cigarette forums.

Multimedia Appendix 3 Keywords for the search for themes within the forums.

## Abbreviations

COREQ: Consolidated Criteria for Reporting Qualitative Research
DSM-5: Diagnostic and Statistical Manual of Mental Disorders, 5th Edition
EVAPE: Evaluation of the Addictive Potential of e-Cigarettes

## Data Availability

The data sets generated during and/or analyzed during the current study are not publicly available to ensure user anonymity but further details may be available from the corresponding author on reasonable request.
