# Supplementary material for: Addictive Potential of e-Cigarettes as Reported in e-Cigarette Online Forums: Netnographic Analysis of Subjective Experiences
Source: J Med Internet Res. 2023 Jan 6;25:e41669. doi: 10.2196/41669 (PMC9862333; doi:10.2196/41669)
Supplement: Multimedia Appendix 2 [file jmir_v25i1e41669_app2.pdf]

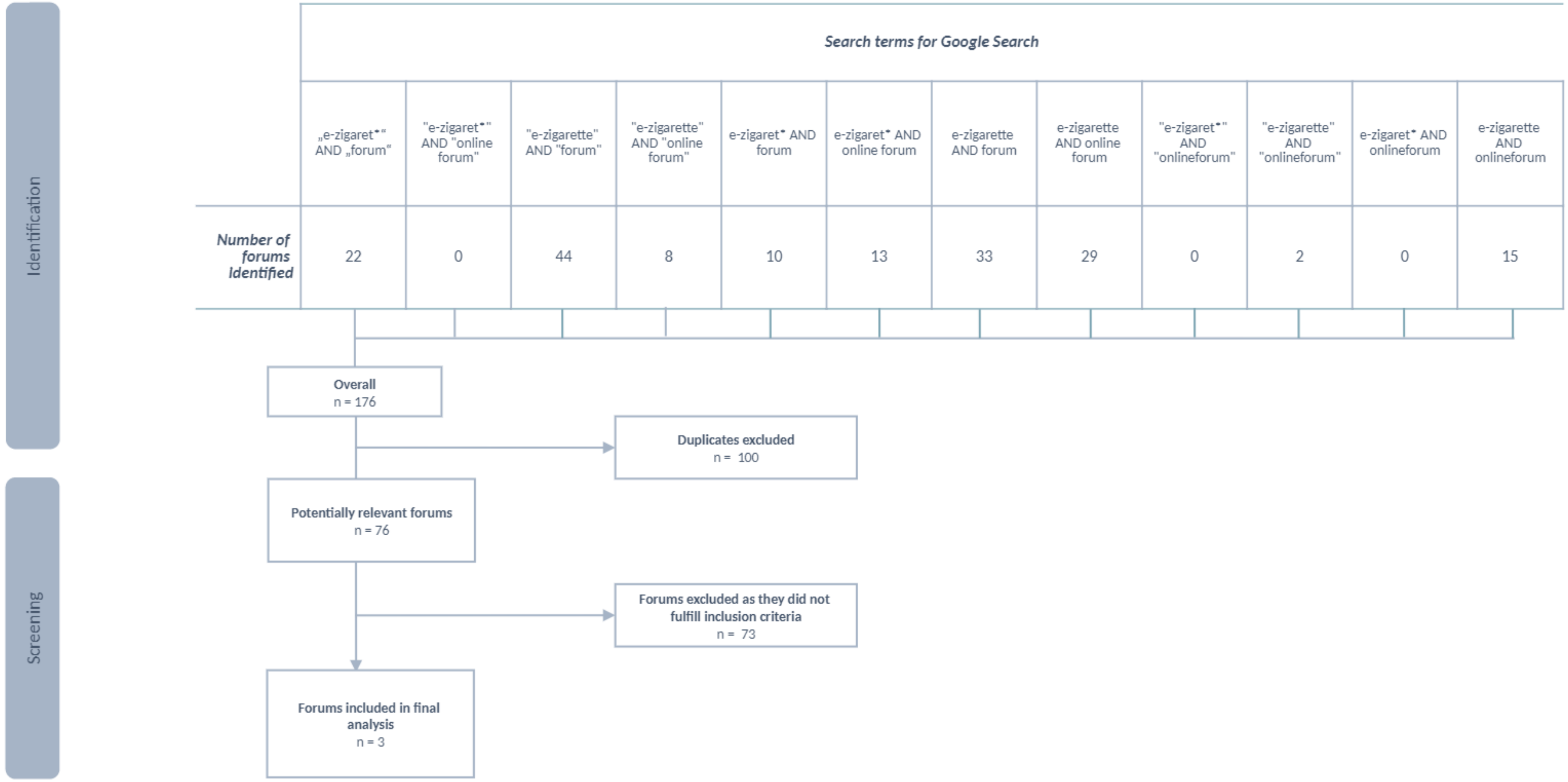
Identification
Screening
Search terms for Google Search
„e-zigaret*" AND „forum"
"e-zigaret*" AND "online forum"
"e-zigarette" AND "forum"
"e-zigarette" AND "online forum"
e-zigaret* AND forum
e-zigaret* AND online forum
e-zigarette AND forum
e-zigarette AND online forum
"e-zigaret*" AND "onlineforum"
"e-zigarette" AND "onlineforum"
e-zigaret* AND onlineforum
e-zigarette AND onlineforum
Number of forums Identified
22
0
44
8
10
13
33
29
0
2
0
15
Overall n = 176
Duplicates excluded n = 100
Potentially relevant forums n = 76
Forums excluded as they did not fulfill inclusion criteria n = 73
Forums included in final analysis n = 3
