# Supplementary material for: Addictive Potential of e-Cigarettes as Reported in e-Cigarette Online Forums: Netnographic Analysis of Subjective Experiences
Source: J Med Internet Res. 2023 Jan 6;25:e41669. doi: 10.2196/41669 (PMC9862333; doi:10.2196/41669)
Supplement: Multimedia Appendix 3 [file jmir_v25i1e41669_app3.pdf]

## Keywords for the search for themes within the forums

| DSM addiction criterion | Keyword German | Keyword English |
|---|---|---|
| **Criterion 1:** E-cigarette is often consumed in larger amounts or for longer than intended. | dampfe immer mehr | vape more and more |
| | Kontrollverlust | loss of control |
| **Criterion 2:** Persistent desire or unsuccessful attempts to reduce or control e-cigarette use. | niedriger dosieren | lowering the dosage |
| | runterdosieren | another word for *lowering the dosage* |
| **Criterion 3:** High time expenditure to obtain, consume, or recover from the effects of e-cigarette use. | am Dauernuckeln | sucking continuously |
| | Dampfsucht | vape addiction |
| **Criterion 4:** Craving or a strong desire to use e-cigarette. | Verlangen | craving |
| | Schmacht | German word for *strong desire* |
| | Drang | urge |
| **Criterion 5:** Repeated e-cigarette use that results in failure to fulfill important responsibilities at work, school, or home (e.g., work disability). | Probleme bei der Arbeit | problems at work |
| | Verpflichtungen bei der Arbeit | obligations at work |
| | Behinderung der Arbeit | obstacle at work |
| | Problem zu Hause | problem at home |
| | Probleme zu Hause | problems at home |
| | Problem daheim | another phrase for *problem at home* |
| | Probleme daheim | another phrase for *problems at home* |
| | Verpflichtungen zu Hause | household chores |
| | Verpflichtungen daheim | another phrase for *household chores* |
| **Criterion 6:** Continued e-cigarette use despite ongoing or repeated social or interpersonal problems caused or exacerbated by the effects of e-cigarette (e.g., arguing with others about e-cigarette use). | provokativ | provocative |
| | dumme Sprüche | silly comments |
| **Criterion 7:** Important social, professional, or recreational activities are abandoned or curtailed because of e-cigarette use. | Kontakt abgebrochen | breaking off contact [with others] |
| **Criterion 8:** Repeated e-cigarette use in situations where use leads to physical danger (e.g., smoking in bed). | körperliche Gefährdung | physical danger |
| | Gefährdung wegen Dampfen | danger due to vaping |
| | gefährliche Situation | dangerous situation |
| **Criterion 9:** Continued e-cigarette use despite knowledge of a persistent or recurring physical or psychological problem that is likely caused or exacerbated by e-cigarettes. | gesundheitliches Problem | health problem |
| | gesundheitliche Probleme | health problems |
| **Criterion 10:** Development of tolerance, defined by any of the following: a) desire for marked increase in dose to bring about a desired effect, | Dosissteigerung | increasing the dosage |
| | mehr Nikotin | more nicotine |

| | | |
|---|---|---|
| b) significantly reduced effect with continued use of the same amount | | |
| **Criterion 11:**<br>Withdrawal symptoms manifested by any of the following:<br>(a) characteristic withdrawal syndrome related to e-cigarettes,<br>b) e-cigarette (or nicotine in the case of nicotine-containing e-cigarettes) is used to relieve or avoid withdrawal symptoms. | Entzugssymptom | withdrawal symptom |
